# Cryptococcus Infection in an Immunocompetent Patient

**DOI:** 10.7759/cureus.27635

**Published:** 2022-08-03

**Authors:** Kofi Seffah, Walter Agyeman, Jennifer L Madeo, Ayesha Ahmad

**Affiliations:** 1 Internal Medicine, Piedmont Athens Regional Medical Center, Athens, USA; 2 Infectious Disease, Piedmont Athens Regional Medical Center, Athens, USA

**Keywords:** cryptococcoma, lumbar punctures, intracranial pressure, embolic phenomena, risk factors, cryptococcal meningitis

## Abstract

Cryptococcal meningitis is a fungal infection of the CNS, generally thought of as an opportunistic infection in those with T-cell immunodeficiencies including AIDS (usually with a CD4 count of less than 100), chronic steroid use, hematological malignancies, and transplant recipients.

It can have irreversible CNS morbidity, including vision loss, intracranial hypertension, and cognitive decline. Diagnosis depends on cerebrospinal fluid (CSF) analysis, in which cultures and cryptococcal antigen are most sensitive. CSF PCR can also be done. Most patients have disseminated disease, and blood cultures are also positive. Outcomes remain guarded, with a poor prognosis (morbidity and high mortality) among survivors. This article presents a case of cryptococcal meningitis in an immunocompetent individual, where absolutely no identifiable risk factor was present.

## Introduction

*Cryptococcus* is an encapsulated yeast found worldwide in the soil, in particular with bird droppings. There are multiple species, but *Cryptococcus neoformans* is most commonly associated with human infection. Transmission is by inhalation. The yeast can remain dormant in the lungs or cause indolent pneumonia in immunocompetent people. In an immunocompromised host, cryptococcus disseminates and has a predilection for the CNS. It is the most common cause of opportunistic yeast meningitis. The most feared sequela is elevated intracranial pressure causing vision loss, neurological deficits, seizures, and a comatose state. In addition, the infection has been associated with cerebral infarcts and space-occupying lesions called cryptococcomas that can exacerbate neurological deficits.

Patients living with HIV (with a CD4 count of less than 100) are at the highest risk for cryptococcal meningitis. There are an estimated one million cases of HIV-associated cryptococcal meningitis globally every year, leading to about 625,000 deaths. The disease is uniformly fatal without treatment. Even with treatment, mortality is estimated to be 20%, mostly due to elevated intracranial pressure. Data on the disease among immunocompetent patients is, however, uncertain, with an estimated 3,200 documented new cases per year in the United States alone. Prognosis is better among immunocompetent people.

Here, we present a case of disseminated *Cryptococcus neoformans* with meningitis in an otherwise healthy female presenting with about one week of persistent headache.

## Case presentation

A 65-year-old female with a past medical history of hypertension, osteoporosis, and schizophrenia was transferred to the hospital after several days of worsening altered mental status/encephalopathy, generalized weakness, and poor appetite at a referring facility. Upon arrival, she was unable to provide any history due to severe lethargy and confusion. She spent a total of 12 days at the referring facility.

The patient was admitted on March 30, 2022. She had no prior history of a rash, injection drug use, immunosuppressive medications, or steroids. She had an unremarkable travel history and had no history of recurrent bacterial, fungal, or viral illness. She had no documented congenital anomalies. A fundoscopic examination showed approximately 2+ disc edema in both eyes with obscuration of the vessels and associated retinal hemorrhages inferior to the disc in the left eye. She, however, had no fever, chills, cough, shortness of breath, neck stiffness, chest pain, or abdominal pain but appeared dehydrated. She was noted to have leukocytosis (Table 1) and hyperchloremia (Table 2). She was placed on piperacillin-tazobactam and intravenous fluids, with an initial diagnosis of a urinary tract infection (UTI), but her cognitive function was noted to progressively worsen.

**Table 1:** Complete blood count at presentation

		Reference range	Units
Hemoglobin (Hb)	12.3	12-14	g/dL
Mean corpuscular volume (MCV)	87.1	80-100	fL
Packed cell volume (PCV)	37	35-45	%
Red cell distribution width (RDW)	16.1	11.6-14.5	%
White blood cell (WBC) count	18.5	4-10.5	/μL
Platelets	419	150-400	/μL
Red blood cell (RBC)	4.25	3.7-5	/μL

**Table 2:** Comprehensive metabolic panel at presentation

		Reference range	Units
Sodium (Na)	143	135-145	mmol/L
Potassium (K)	3.7	3.5-5	mmol/L
Chloride (Cl)	109	98-108	mmol/L
Bicarbonate	25	22-28	mmol/L
Blood urea nitrogen (BUN)	11	5-20	mg/dL
Creatinine	0.65	0.6-1.2	mg/dL
EGFR	>60	>60	mL/minute/1.73 m^2^

The lumbar puncture performed noted cerebrospinal fluid (CSF) glucose of 20 mg/dL, blood glucose of 106 mg/dL, CSF protein of 115 mg/dL, CSF RBCs of 7/mm^3^, nucleated cells of 54, 16% neutrophil, and 84% lymphocytes. The CSF grossly was colorless and clear. CSF PCR for *Cryptococcus neoformans* was positive. Opening pressure was not performed. Five days into her admission, the patient tested positive for fungemia. She was started on micafungin on account of her fungemia.

Repeat culture showed persistence of her fungemia, and *Cryptococcus neoformans* was identified as the causative agent. Micafungin was deemed inappropriate in managing this infection, and the patient was subsequently switched to liposomal amphotericin B with flucytosine. Blood cultures from February 7, 2022, returned negative, and the patient remained negative throughout the duration of her admission. The patient was referred for higher care in February 10.

The patient arrived at the hospital in February 11. The Infectious Disease unit was notified, and amphotericin B and flucytosine were restarted. Lumbar puncture repeated on arrival confirmed the diagnosis of cryptococcal meningitis.

**Figure 1:**
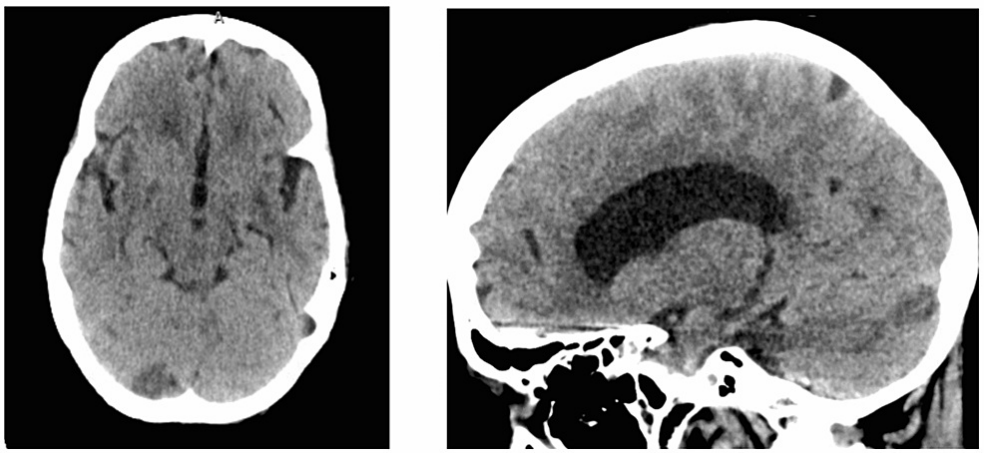
Head CT on February 16, 2022 Impression: Hypodensity in the posterior medial portion of the right occipital lobe, suggesting an acute to subacute infarct.

**Figure 2:**
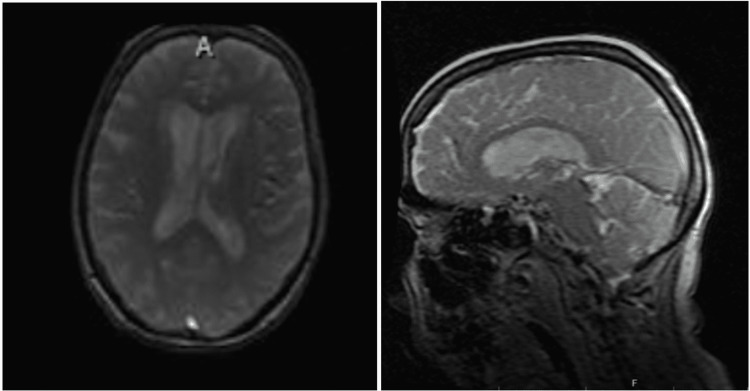
MRI of the brain on February 17, 2022 Restricted diffusion is noted in the medial aspect of the right cerebellar hemisphere likely representing an acute infarct. Additional acute slit-like infarcts are noted in the bilateral cerebellar hemispheres. There is a prominent motion artifact, but there appears to be restricted diffusion at the gray-white junction throughout the bilateral cerebral hemispheres, likely representing acute infarcts.

**Figure 3:**
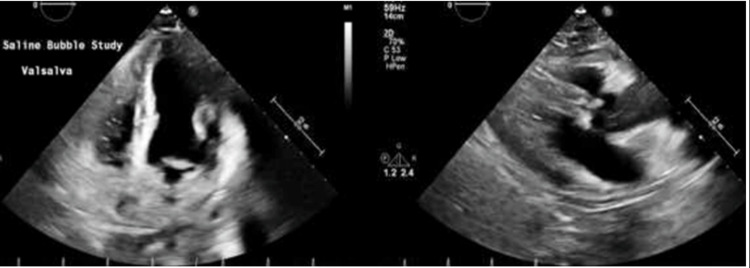
Transthoracic echocardiogram on February 17, 2022 Conclusion: LVEF is 70%, grade I mild diastolic dysfunction, no evidence of patent foramen ovale by agitated saline contrast, mildly elevated RV systolic pressure, and no significant pericardial effusion.

The patient’s mental status was noted to wane after two days on admission, despite continued antifungal treatment. A neurosurgery review recommended a CT scan of the brain (Figure 1), and the findings suggested an acute ischemic event in the territory of the cerebellum. This was confirmed by an MRI of the brain (Figure 2). A thorough workup for the source of the stroke, however, ruled out the usual sources, with patent carotids on Doppler ultrasound, no patent foramen ovale or ventricular septal defect, and no arrhythmias or vegetations involving the cardiac valves (Figure 3). In addition, her stroke was not enough to explain her fluctuating mental status. Although there were ischemic changes and clear sepsis, it was still unclear at this point if the patient would benefit from lumbar punctures.

Owing to deteriorating mental status and notably requiring standard care for C meningitides, the decision for subsequent serial therapeutic lumbar punctures was made. Opening pressures were notably high, at 30 cmH20, following the first tap on February 17, 2022, and progressively noted to increase in the subsequent tap to 50 cmH20 on February 21, 2022. Her mental status was noted to improve transiently with each spinal tap, but this was seemingly short-lived, at about 6-hour duration, following which she would be noted to be confused and barely arousable. In February 18, the patient was noted to have seizure-like activity, characterized by staring and generalized convulsions and for which levetiracetam was commenced. She continued to have seizures (February 19, 2022, and February 23, 2022) despite anti-seizure prophylaxis, in conjunction with optimal antifungal treatment and serial lumbar punctures.

The patient was transferred to the ICU on February 21, 2022, for a higher level of care.

Although blood cultures were negative before her transfer to the hospital, a positive CSF culture was noted on February 23, 2022, about 10 days after the onset of sensitivity-proven antifungals. CSF PCR was positive with each draw. A lumbar drain was placed in February 28. Drainage was, however, discontinued on March 6, 2022, and the drain was clamped and removed in March 13. Awareness and response to stimuli continued to decline despite these measures until she was noted to only be minimally responsive to extreme pain about two weeks into the onset of treatment. While antifungals were continued, the patient was placed in palliative care, as her prognosis remained guarded. She remained barely responsive by the time of transfer out to the hospice home on March 25, 2022. Her GCS remained less than 8 on discharge.

## Discussion

Cryptococcal meningitis has previously been associated with immunosuppression [1]. It has particularly been linked with HIV infection as an opportunistic infection [2]. It is a major cause of morbidity in people living with HIV/AIDS, with about 220,000 cases of cryptococcal meningitis occurring worldwide each year [2]. Notably, while the prevalence of the disease is estimated at about one million cases worldwide, it is widely considered to occur almost exclusively in immunosuppressed patients [3]. *Cryptococcus neoformans* infections are still rare among people who have healthy immune systems [3]. Conditions associated with immunosuppression include leukemia, diabetes, transplant recipient on immunosuppressants, autoimmune disease, chronic steroid use, and congenital B-cell and T-cell defects [4,5]. However, there is a trend showing a shift from HIV-positive and solid organ transplant patients toward non-HIV and non-transplant patients [4].

Our patient did not have any of the conditions listed above. She did not live in a congregate setting and had not been exposed to any individual with a chronic infectious disease or been diagnosed with a congenital defect. She did not have a history of recurrent infectious illness, which would suggest immunosuppression. Although she was COVID-19-positive, this has not been associated with a chronic immunosuppressive state [6]. In addition, she only tested positive for COVID-19 and did not have physical findings (such as an increased need for oxygen) or radiologic changes that would implicate pulmonary infection.

Cryptococcus is an environmental microbe, which may exist in the respiratory tract of humans and on soil and plants [7,8]. By definition, as an opportunistic infection, it becomes active when the immune system of its host is compromised [9]. The particular source of our patient’s microbe cannot be easily identified. Unwashed raw fruit, bird droppings, trunk hollows of trees, bat guano, and exposure to eucalyptus have previously been documented as sources [10-12]. Her history was not suggestive of recent or previous chronic exposure to any of these factors. It is intriguing that *Cryptococcus* should emerge as the cause for her presentation, thus posing a relevant question that needs further probing. We seek to present findings we had, noting no previously clear trend to the presentation of this infection in the immunocompetent, and hope to add to the emerging body of knowledge for potential prevention in the future. We also focus on atypical/unusual findings associated with the presentation, in order to create more awareness of the condition for earlier diagnosis.

It must be stressed that there have been documented cases of cryptococcal meningitis in immunocompetent patients, which also did not identify any clear exposure pattern or underlying genetic defects that may have predisposed to the infection [13,14].

When common sources of suppressed immunity cannot be identified, one must assume that conditions such as diabetes, chronic liver disease, and chronic kidney disease may be at play and may likely be responsible. The overall mortality from cryptococcal meningitis is documented to be between 12% and 25% in the immunocompetent, owing in part to atypical/insidious presentation [15,16].

## Conclusions

Cryptococcal meningitis, even among the immunosuppressed, is a rare condition. There is growing evidence that the condition may be found in the immunocompetent. In the absence of organ transplants, chronic steroid use, and conditions such as HIV/AIDS, less prominent causes of suppressed immunity must be accounted for, including diabetes and chronic liver and kidney diseases. In the absence of all these, there is room for more study in this regard, concerning potential host and environmental factors, yet undiscovered, that may play a major role in understanding the pathophysiology of this disease entity.
